# Prohibition

**Published:** 1887-10

**Authors:** 


### PROHIBITION.

There is a good deal of present agitation looking to the enactment of state laws restricting the retail traffic in spirituous liquors. Such laws have been passed and gone into effect in a number of states, notably, Maine and Kansas, and the friends of such measures claim that great good has resulted from them.

It is a fact too notorious for argument, that by far, the most prolific source of crime and misery which mar our civilization, and corrupt the very fountain of national prosperity, is to be found in the free use of intoxicating liquors, and any order of things which would serve to root out this evil, effectually and forever, would bring in its enforcement, unspeakable blessings to countless homes where now only discord and desolation prevail. But, unfortunately, there is a want of harmony, or at least, of singleness of purpose, amongst the various organizations formed to effect a reform, in respect to which there is such urgent need. Some advocate total prohibition, others, a qualified or restricted use of spirituous liquors as a beverage, whilst others still, confine their efforts to the advocacy of a system of "high license," which would tend to cut off many of the least pretentious groggeries, and confine the traffic to a class of better conditioned servitors.

There is in New York City, an incorporated "Society for the Prevention of Crime," which is presided over by a distinguished clergyman of the old school, whose name has prominently figured in many benevolent enterprises. One would naturally suppose that such an organization, properly equipped, would find no lack of employment in the vicinity of its headquarters, wherever that might be in the great city to which its efforts are mainly confined; but, so far as we have been able to learn, the sole endeavor of this society has been to prevent the retailing of spirituous liquors by unlicensed venders, and its sale under license at unauthorized hours. This society has been in operation for some years. Its officers are able men and some of them are zealous and active in their work; it spends a good deal of money, tries to make police officers do their duty—no inconsiderable task—succeeds, now and then, in rooting out some villainous dive which is at once re-established, but does no practical, permanent good, and this seems to be the case with nearly all such half-way attempts.

It has been stated by those whose opportunities of knowing are the best, that more than ninety per cent. of the crime committed in this country, is due to intoxication, and the moral depravity attendant upon the habitual use of strong drink. If this be so, as it doubtless is, no one thing would add so much to the prosperity and good order of the commonwealth, as the total prohibition of the use of intoxicants as a beverage.

Let us take New York City as an illustration: There is gathered within its territorial limits, a population of a million and a half. It has been estimated that $250,000 a day are spent there for drink, making $1,500,000 a week and $75,000,000 in a single year. Of this vast sum it may not be unreasonably inferred that one-fifth or $15,000,000 is squandered by the working classes whose families are, in very many instances, put to great straits for the actual necessaries of life.

What is true of New York City, is true of the whole country. The enormity of this evil, which, like a dry rot, is sapping the foundations of government, cannot be over-estimated. We have only a side view of it in being brought face to face with the figures. In the same ratio the sixty millions population of the United States spend three hundred millions for drink every year, a sum more than sufficient to meet all the requirements of our national government.

When we add to this the crime and misery to which this senseless habit gives rise, the picture is one which it is impossible to look upon with composure. Where then is the remedy to be found for this wide-spread evil; this multiplier of prisons and poor houses, and waster of populations? Is it in state legislation? That has been tried to little or no purpose. Is it in the organization of total abstinence societies? Such have existed indefinitely with no sign of the end so devoutly to be wished.

How does the general government stand upon this question? in other words, what efforts have the people, through their representatives in congress, made to overcome this enemy of their homes and firesides? Has a single move been made looking to its destruction? The truth is, that the government is largely sustained by encouraging and taxing this nefarious business.

If it were possible to put an end to it, what a prosperous and happy people we might be, and *it is possible.* Let congress prohibit the manufacture of cereals into whisky. If this prolific source of crime is ever to be effectually eradicated, we must strike at its root. No amount of trimming at the extremes, pinching off buds and severing branches, will suffice. What a saving of life, of property, of morals, of comfort and of happiness, would result from such a measure.

Something over a quarter of a century ago, the Chinese government made an effort to put a stop to the opium habit, which was demoralizing her people and weakening the nation, by prohibiting the importation of opium into the empire, but the effort failed by reason of the ability of England to enforce the opium traffic upon them, and America, is in some small degree, made to suffer from it now. But there is no fear of any outside pressure being brought to bear upon us. We have always been left to regulate our domestic concerns in our own way. Should government enact that no beverage should be produced from the distillation of grain or cereals, anywhere within its territorial limits, it would come far short of provoking a revolution, and, although the fastnesses of the Alleghanies might afford a hiding place for illicit distillation, it would be of small moment and soon die out. Then, many prison houses would be tenantless, poor houses abandoned, and fierce and uncontrollable dissipation give way to sober industry and the beatification of home, and government would cease to be a conservator of its own enfeeblement and disintegration. There are two present communities of workingmen whose prosperity is of the highest. One is located in the east near Hartford, the other, in the west near Chicago, and both are types of what the whole country might be under a wise and temperate system. Instead of groggeries there are libraries of useful books ; in the place of idle resorts there is the reading-room well supplied with current intelligence from all over the world, where men and women meet upon equal terms and seek the higher levels of thought and action. What is true of these communities might be true of the whole country, under equally wholesome regulations.

Let us hope for the coming of the day.

